# Reply to Frański, R. Comment on “Hu et al. Determination of 2-Pentanol Enantiomers via Chiral GC-MS and Its Sensory Evaluation in Baijiu. *Foods* 2022, *11*, 2584”

**DOI:** 10.3390/foods12071450

**Published:** 2023-03-29

**Authors:** Lisha Hu, Yifeng Dai

**Affiliations:** School of Liquor and Food Engineering, Guizhou University, Guiyang 550025, China

The comments [1] concerning the article entitled “Determination of 2-pentanol enantiomers via chiral GC-MS and its sensory evaluation in Baijiu” [2] are very valuable and helpful for us. 

We indeed miswrote the characteristic ions of 2-pentanol, but our qualitative and quantitative experiments were based on the standards of the enantiomers of 2-pentanol, which were (*R*)-2-pentanol and (*S*)-2-pentanol. The ions characteristic of 2-pentanol are at *m*/*z* 45, 55, 43, and 73, and the base peak is always that at *m*/*z* 45, as you mentioned. Here are the reasons why we made such an awkward mistake. At first, we did not write the characteristic ions of 2-pentanol in the original manuscript that we submitted to the journal *Foods*. When we received the reviewers’ comments from the editor, we added the characteristic ions according to the reviewers’ suggestion during the revision period. Unfortunately, we did that in a hurry in order to submit the revised manuscript sooner and clicked on the wrong chromatography and wrote the wrong ions in the revised manuscript. However, the qualitative and quantitative experiments that we had conducted on Baijiu samples were all based on the standards of (*R*)-2-pentanol and (*S*)-2-pentanol.

In order to confirm the qualitative results of (*R*)-2-pentanol and (*S*)-2-pentanol, we repeated some experiments of some samples by LLE-GC-MS with the same conditions as we described in the published article [2], with the same type of column but a new one(CYCLOSIL-B (30 m × 0.25 mm × 0.25 µm, Agilent Technologies Ltd., Santa Clara, CA, USA). Figure 1 showed a few exemplary chromatograms and mass spectra. The chromatogram of enantiomeric separation and mass spectra of the standard of 2-pentanol were shown in Figure 1a–c. Enantiomeric separation chromatograms of 2-pentanol in representative Baijiu samples, such as WLY, SJF, and YHMZL were shown in Figure 1d,g,j, respectively. Additionally, the exemplary mass spectra of (*R*)-2-pentanol and (*S*)-2-pentanol for the WLY sample were shown in Figure 1e,f, respectively. The exemplary mass spectra of (*R*)-2-pentanol and (*S*)-2-pentanol for the SJF samples were shown in Figure 1h,i, respectively. The exemplary mass spectra of (*R*)-2-pentanol and (*S*)-2-pentanol for the YHMZL sample were shown in Figure 1k,l, respectively. It was clearly the case that the ions characteristic of both enantiomers of 2-pentanol were 45, 55, 43, and 73 in the Baijiu samples. Thank you very much for your careful review.

2-Pentanol was mentioned as being one of the important alcohols in Moutai [3]:

2-Pentanol was listed in the article by Zhu et al. [3] in Table 4 “Some important alcohols identified in Moutai liquor sample 2” on page 346, where it was considered to be one of the important alcohols identified in the Moutai liquor sample 2 by Zhu et al.

2-Pentanol was mentioned in Yanghe Daqu as one of the potentially important alcohols [4]:

Fan et al. [4] mentioned that in the fourth paragraph on page 7934: 

On the basis of the FD values detected on a DB-Wax column (Table 1 [4]), the potentially important alcohols were 2-butanol, 3-methylbutanol, 2-pentanol, and 1-hexanol (FD ≥ 32). These alcohols impart fruity, floral, green, and alcoholic odors. These alcohols had high FD values in aged liquor and low FD values in young liquor. Among them, 3-methylbutanol and 2-pentanol had FD values more than 256 in aged liquor and less than 4 in young liquors. 

Furthermore, on page 7937, the author mentioned that “Ethyl 2-methylbutanoate and ethyl 4-methylpentanoate were very important to Yanghe Daqu liquor aroma because of their high FD values (FD ≥ 256)”. Since 2-pentanol was reported to have FD values higher than 256 (Table 1) [4] in aged Yanghe Daqu liquor, it may be very important to Yanghe Daqu’s liquor aroma. However, this reference [4] was missing in our paper.

2-Pentanol was mentioned as being one of the potentially important alcohols in Wuliangye [5]:

Fan et al. [6] determined FD ≥ 8 for 2-pentanol in the Acidic/Water-Soluble fraction in Wuliangye, which was listed in Table 2 [6] on page 2699. Niu et al. [5] mentioned in the “RESULT AND DISCUSSION” section that “On the basis of the FD values detected on a Innowax-Wax column, potentially most important alcohols were 2-pentanol, 1-butanol, 2-phenylethanol, 1-pentanol, 3-methylbutanol, 2-ethyl-1-hexanol, furfuryl alcohol and 1-hexanol (FD ≥ 16)”. Therefore, 2-pentanol was considered to be one of the potentially most important alcohols by Niu et al. [5].

In summary, we wrote that “2-Pentanol … is considered to be an important volatile substance in Moutai, Yanghe Daqu, [and] Wuliangye…”. However, from a rigorous academic standpoint, it would be better to state that 2-pentanol is considered to be an important volatile substance in Moutai, and a potentially important volatile substance in Yanghe Daqu and Wuliangye.

Regarding the mentioned sentence, which can be found on page 1 (“As shown in Table 1 [2], chiral compounds in alcoholic beverages usually used direct injection (DI), liquid-liquid extraction (LLE) and solid-phase microextraction (SPME), et al. …”), it is a comment on the current pre-treatment methods for chiral compounds in alcoholic beverages.

As per the second sentence, from page 3 (“Take 5 µL of 2-pentanol racemate with anhydrous ethanol …”), it means that the standard of 2-pentanol racemate was diluted to a low concentration. Here, what we described was the standard of 2-pentanol, not the 2-pentanol in Baijiu samples. The purpose of this was not only to avoid a high concentration (purity) of the standard of 2-pentanol racemate remaining in the chiral columns (leading to residue in columns), but also to get a better resolution of the chromatographic separation. We wanted to first separate the standard of 2-pentanol racemate using different chiral columns (stationary phases) to get a higher resolution, then separate the 2-pentanol enantiomers in Baijiu based on the above higher resolution chiral column. Therefore, the two sentences in the paper are not conflicting or awkward.

Thank you again for your careful and valuable comments. We will check more carefully on what we write in our future work.

## Figures and Tables

**Figure 1:**
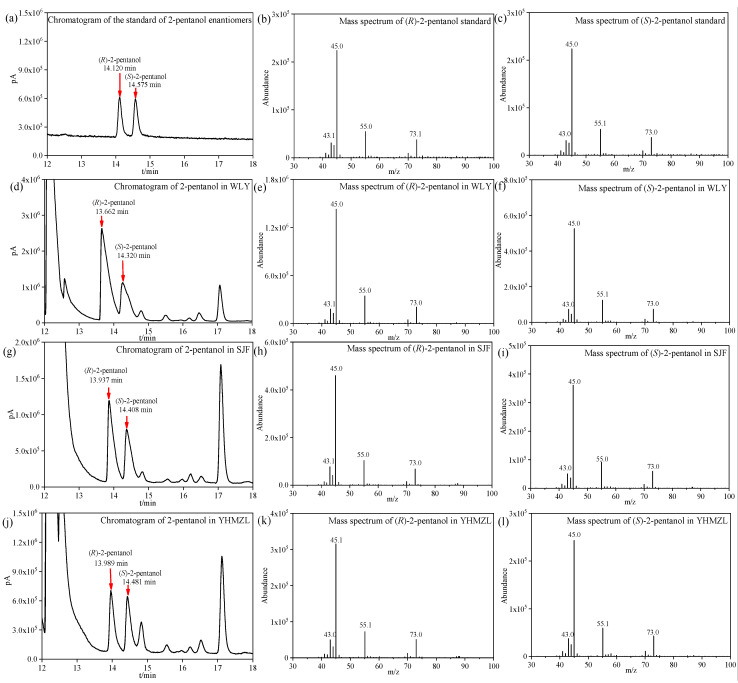
Enantiomeric separation chromatograms and exemplary mass spectra of 2-pentanol in representative Baijiu samples: (**a**–**c**): the standard of 2-pentanol enantiomers; (**d**–**f**): WLY sample; (**g**–**i**): SJF sample; (**j**–**l**): YHMZL sample.

## Data Availability

No new data were created.
